# Estimación de la demanda de medicamentos antichagásicos: una contribución para el acceso en América Latina

**DOI:** 10.26633/RPSP.2017.45

**Published:** 2017-04-14

**Authors:** Gabriela Costa Chaves, Mariana Abi-Saab Arrieche, Joelle Rode, Daniel Mechali, Priscileyne Ouverney Reis, Renato Vieira Alves, Eric Stobbaerts, Nora Girón Aguilar, Isabela Ribeiro

**Affiliations:** 1 Escola Nacional de Saúde Pública Sergio Arouca Fundação Oswaldo Cruz Rio de Janeiro Brasil Escola Nacional de Saúde Pública Sergio Arouca, Fundação Oswaldo Cruz, Rio de Janeiro Brasil.; 2 Iniciativa Medicamentos para Enfermedades Olvidadas (DNDi, por sus siglas en inglés) Iniciativa Medicamentos para Enfermedades Olvidadas (DNDi, por sus siglas en inglés) Rio de Janeiro Brasil Iniciativa Medicamentos para Enfermedades Olvidadas (DNDi, por sus siglas en inglés), Rio de Janeiro, Brasil.; 3 Governo do Distrito Federal, Secretaria de Estado de Saúde do Distrito Federal Subsecretaria de Vigilância à Saúde Brasília Brasil Governo do Distrito Federal, Secretaria de Estado de Saúde do Distrito Federal, Subsecretaria de Vigilância à Saúde, Brasília, Brasil.; 4 Unidade Técnica de Vigilância das Doenças de Transmissão Vetorial, Secretaria de Vigilância em Saúde Ministério da Saúde do Brasil Brasília Brasil Unidade Técnica de Vigilância das Doenças de Transmissão Vetorial, Secretaria de Vigilância em Saúde, Ministério da Saúde do Brasil, Brasília, Brasil.; 5 Organización Panamericana de la Salud Organización Panamericana de la Salud Honduras Organización Panamericana de la Salud, Honduras.; † Fallecido el 7 de abril de 2016. Fallecido el 7 de abril de 2016. Fallecido el 7 de abril de 2016.

**Keywords:** Enfermedades desatendidas, enfermedad de Chagas, equidad en el acceso, medicamentos esenciales, Neglected diseases, Chagas disease, equity in access, drugs, essential, Doenças negligenciadas, doença de chagas, equidade no acesso, medicamentos esenciais

## Abstract

***Objetivo*.:**

Describir una herramienta de estimación de la demanda de benznidazol y nifurtimox para la enfermedad de Chagas y relatar su aplicación en un conjunto de países de América Latina.

***Métodos*.:**

El proyecto se desarrolló en las siguientes etapas: 1) elaboración de una herramienta y definición de las variables de evaluación y decisión para estimación de la demanda, 2) recolección de datos a partir de un cuestionario a representantes de programas de control, complementado con datos de la literatura, 3) presentación, validación y adaptación de la herramienta por representantes de los programas de control, con la intención de planificar la adquisición de medicamentos para los años de 2012 y 2013, y 4) análisis complementar de los datos obtenidos, en especial benznidazol, y comparación de las estimaciones de los países.

***Resultados*.:**

Catorce países endémicos de América Latina participaron de la tercera etapa, con definición de una estimación consolidada. El número de tratamientos estimados a partir del número de comprimidos por tratamiento establecido en el esquema de referencia resultó ser de 867 en menores de un año, 2 042 835 en el grupo de 1 a 15 años, 2 028 en el grupo de 15 a 20 años y de 10 248 en adultos mayores de 20 años. Este cuantitativo representa la posibilidad de tratar menos de 1% de las personas con indicación para tratamiento con benznidazol.

**Conclusiones.:**

El desarrollo y el uso sistemático de herramientas de gestión de la demanda pueden jugar un papel clave en el apoyo al acceso a los medicamentos en la enfermedad de Chagas. Existe una brecha significativa entre las previsiones de demanda de medicamentos y estimaciones actuales de las tasas de prevalencia.

## Abstract (en)

***Objective*.:**

Describe a tool to estimate demand for benznidazole and nifurtimox to treat Chagas disease, and report on its implementation in a group of Latin American countries.

***Methods*.:**

The project was carried out in the following stages: 1) development of a tool to estimate demand, and definition of the evaluation and decision variables to estimate demand 2) data collection via a questionnaire completed by representatives of control programs, complemented with data from the literature; 3) presentation of the tool, followed by validation, and adaptation by representatives of the control programs in order to plan drug procurement for 2012 and 2013; and 4) further analysis of the obtained data, especially regarding benznidazole, and comparison of country estimates.

Fourteen endemic countries of Latin America took part in the third stage, and a consolidated estimate was made. The number of estimated treatments, based on the number of tablets per treatment established in the regimen of reference was: 867 in the group under 1 year of age; 2 042 835 in the group from 1 to 15 years old; 2 028 in the group from 15 to 20 years old; and 10 248 in adults over 20. This means that it is possible to provide benznidazole to less than 1% of people for whom treatment is indicated.

***Conclusions*.:**

The development and systematic use of demand management tools can play a key role in helping to provide access to the anti-Chagas drugs. There is a significant gap between the projected demand for drugs and current estimates of prevalence rates.

## RESUMO

Descrever uma ferramenta de estimativa da demanda de benznidazol e nifurtimox para a doença de Chagas e descrever a sua aplicação em um conjunto de países da América Latina.

O projeto foi desenvolvido nas seguintes etapas: 1) elaboração de uma ferramenta e definição das variáveis de avaliação e decisão para a estimativa da demanda, 2) compilação de dados a partir de um questionário apresentado a representantes de programas de controle, complementado com dados da literatura, 3) apresentação, validação e adaptação da ferramenta por representantes dos programas de controle, com a intenção de planejar a aquisição de medicamentos para os anos de 2012 e 2013 e 4) análise complementar dos dados obtidos, especialmente sobre o benznidazol, e comparação das estimativas nos países.

Quatorze países endêmicos da América Latina participaram da terceira etapa, sendo definida uma estimativa consolidada. O número de tratamentos estimados a partir do número de comprimidos por tratamento estabelecido no regime de referência foi de 867 em pacientes menores de um ano, 2.042.835 no grupo com idade entre 1 e 15 anos, 2.028 no grupo entre 15 e 20 anos e 10.248 em adultos maiores de 20 anos. Esta quantidade representa a possibilidade de tratar menos de 1% das pessoas com indicação para tratamento com benznidazol.

***Conclusões*.:**

O desenvolvimento e o uso sistemático de ferramentas de gestão da demanda podem ser fundamentais para apoiar o acesso aos medicamentos para a doença de Chagas. Existe uma disparidade significativa entre as previsões de demanda de medicamentos e as estimativas atuais das taxas de prevalência.

La enfermedad de Chagas fue descrita por primera vez hace 107 años y es endémica en 21 países de América Latina (1). La región representa 78,35% de la carga global anual de la enfermedad y a cada año surgen cerca de 55 000 casos nuevos (1, 2). Al mismo tiempo, la enfermedad presenta un número creciente de personas que viven con la infección en los países desarrollados, originalmente no endémicos para la transmisión vectorial, en función de los flujos migratorios entre países (3–5).

Según la Organización Mundial de la Salud (OMS), el riesgo de transmisión se ha reducido en la región de las Américas gracias a la adopción de medidas de control vectorial y transfusiones de sangre seguras, lo cual originó una disminución estimada de personas infectadas de 20 millones en 1981 para 10 millones en 2009 (1) y 5,6 millones en 2015 (6). A pesar de lo antes dicho, la enfermedad de Chagas continúa siendo una enfermedad de alto impacto, responsable por más de 10 000 muertes al año (1) y significativa morbilidad.

Para los sistemas de salud de los países endémicos, una persona infectada con la enfermedad de Chagas puede representar un costo de USD 3 456 a lo largo de su vida, en cuanto a asistencia médica se refiere. De esta manera, pierde lo equivalente a más de tres años de vida productiva en función a su afección e incapacidad (7).

Los dos medicamentos disponibles para el control etiológico de la enfermedad de Chagas son el benznidazol (producido por el Laboratorio Farmacéutico de Pernambuco, LAFEPE, en Brasil y ELEA® en Argentina) y el nifurtimox (producido por Bayer® en El Salvador), como primera y segunda líneas, respectivamente, del tratamiento en la mayoría de los países. Una de las limitaciones relacionadas al benznidazol fue que, hasta 2012, solo estuvo disponible en una presentación de tabletas de 100 mg. Si se considera que la dosis se calcula según el peso del individuo, el tratamiento en los niños requería triturarlos comprimidos o fraccionarlos hasta en ocho partes. Esto colocaba a este grupo de pacientes en potencial riesgo de sobredosis o subdosis (8).

A partir de 2009, año del centenario del descubrimiento de la enfermedad de Chagas, resoluciones específicas aprobadas por los países en los Consejos Directivos de la Organización Panamericana de la Salud (OPS) (CD 49.09 y CD 50.16) y en la Asamblea Mundial de Salud de la OMS (WHA 63.20) (9–11), reconocieron acciones de diagnóstico y tratamiento como componentes esenciales del abordaje a la enfermedad.

Igualmente, a partir de ese año, como resultado del aumento de las evidencias científicas (12–14) y gracias a las campañas para ampliación del acceso al diagnóstico y tratamiento para la enfermedad de Chagas (15, 16), se observó un ligero crecimiento de la demanda de benznidazol y nifurtimox (2, 17).

Más recientemente, los estudios de Molina y colaboradores (18) y el estudio para evaluación del E1224 (profármaco del ravuconazol) y el benznidazol (19) demostraron una respuesta parasitológica rápida y sostenida en pacientes adultos con enfermedad de Chagas crónica. Por otra parte, los resultados recientemente publicados del estudio BENEFIT (20) indicaron que el tratamiento con benznidazol en personas con enfermedades cardíacas no altera la progresión de la enfermedad, lo que refuerza la importancia del diagnóstico temprano para asegurar el tratamiento oportuno (21).

El acceso a medicamentos de enfermedades desatendidas como la enfermedad de Chagas no puede ser visto como un desafío aislado, sino que debe ser colocado en el contexto del acceso a los servicios de salud. El acceso de los pacientes que viven con la enfermedad de Chagas depende no solo de la oferta de insumos para el tratamiento y diagnóstico, sino también de financiamiento sostenible, de la existencia de una red de servicios de salud confiables y accesibles geográficamente y de un sistema eficiente de la planificación y gestión del abastecimiento (22, 23).

Ampliar la demanda en el escenario de las enfermedades desatendidas significa enfrentar una oferta bastante específica ya que, en la mayoría de los casos, los medicamentos existentes son de fuente única o provienen de pocas fuentes. Esto hace que cualquier variación en la demanda puede afectar la capacidad de respuesta por parte del productor que, a su vez, tendrá consecuencias en la capacidad de respuesta de los sistemas de salud frente a estas enfermedades (24).

Es en este sentido que el acceso a medicamentos para enfermedades desatendidas enfrenta una serie de otras barreras complejas, frente a las cuales la estimación de la demanda de medicamentos puede tener un papel clave (25). Esto se debe que, en primer lugar y desde la perspectiva del productor, se posibilita la planificación de la producción y el manejo de las relaciones con los demás proveedores de la cadena productiva, tal como la producción del principio activo. En segundo lugar, debido a que en la perspectiva de los programas de diagnóstico y tratamiento (gobiernos, organizaciones, instituciones internacionales y otros) tiene el papel de posibilitar la planificación presupuestaria y de la cadena de distribución y logística, para garantizar el cumplimiento de los compromisos en relación a las acciones de diagnóstico y tratamiento y diseñar las políticas necesarias para una expansión de estas acciones.

En el caso de la enfermedad de Chagas, hasta el año 2012 existía un solo productor de benznidazol. El LAFEPE (Brasil) recibió la transferencia de tecnología de la farmacéutica suiza Roche® (26). El período entre el compromiso de ampliación de acceso al tratamiento por parte de los gobiernos y la necesidad del laboratorio de tener una nueva fuente de ingrediente farmacéutico activo (IFA) para producir benznidazol, unido al vencimiento de los últimos lotes del medicamento de Laboratorios Roche®, generó un escenario de desabastecimiento en 2011 (27).

Aún antes de 2009, se realizaron esfuerzos regionales con el objetivo de estimar la demanda de medicamentos antichagásicos (2). Con la crisis de desabastecimiento en 2011 (27, 28), los países de la región indicaron la necesidad de contar con herramientas y capacitación para realizar dicha estimación (29).

En este contexto, el presente proyecto tuvo como objetivo desarrollar una herramienta de estimación de demanda de benznidazol y nifurtimox para la enfermedad de Chagas y relatar su aplicación en un conjunto de países de América Latina.

## METODOLOGÍA

**FIGURA 1.**
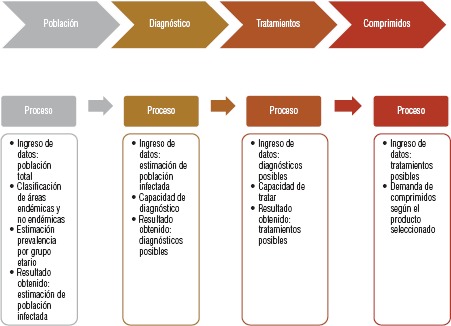
Procesos que intervienen en el desarrollo de la previsión de la demanda y su respectivo cálculo.

Atendiendo al pedido de los países de la región, *Iniciativa* Medicamentos para Enfermedades Olvidadas (DND*i*, por sus siglas en inglés), Médicos Sin Fronteras (MSF) y la OPS desarrollaron una herramienta de estimación de demanda de los medicamentos antichagásicos (17). A partir de discusiones con expertos, se definieron las variables de evaluación y decisión para estimación de la demanda: población total y por grupo etario, clasificación de áreas endémicas y no endémicas, prevalencia de la enfermedad de Chagas, número de puntos de atención médica y diagnóstico, e informaciones sobre programas especiales, aumento de profesionales involucrados, aumento de nuevos centros de atención (figura 1).

Una vez diseñada la herramienta, se inició el proceso de presentación, aplicación y validación en su versión disponible para la época (2.0 – 2012). Se realizó una recolección inicial de datos a partir de un cuestionario entregado a los representantes de los programas de control, complementado con datos de la literatura.

En julio de 2012, el Ministerio de Salud de Brasil, la OPS, DND*i* y MSF realizaron un taller con los países de la región latinoamericana para apropiarse del uso de la herramienta y establecer una agenda de trabajo conjunta para la planificación de la adquisición de benznidazol y nifurtimox en 2012 y 2013. Participaron 14 países endémicos de la región: Argentina, Brasil, Bolivia, Colombia, Chile, Ecuador, El Salvador, Guatemala, Honduras, México, Nicaragua, Panamá, Paraguay y Perú. Se contó con la representación de Médicos Sin Fronteras, organización que para la época desarro-llaba proyectos integrales de diagnóstico y tratamiento para la enfermedad de Chagas en Bolivia y Paraguay. Se solicitó con antecedencia que los países llevaran los datos epidemiológicos, histórico de compras y de tratamientos para viabilizar el cálculo de demanda con la herramienta durante el taller.

Se realizaron algunos cambios en la herramienta a partir de su presentación y aplicación por los representantes de los programas de control, tal como la incorporación del histórico de compras y del histórico de personas tratadas como alternativa para el cálculo de la estimación. Se consideró también el lanzamiento de nuevas presentaciones de benznidazol: 50 mg y 12,5 mg (30, 31), exigiendo con esto la necesidad de seleccionar un mix de productos, además de considerar franjas etarias específicas.

***Fuente:*** adaptado de DNDi, OPS, MSF (2013).

En el cuadro 1 se presenta un resumen de las adaptaciones de la herramienta. La última versión de la herramienta (versión 3.0) está disponible en línea (http://www.dndial.org/chagasforecasting/Demand-ForecastingChagasIII_FinalJulho2013.xlsm) junto con el Manual del Usuario (http://www.dndial.org/chagasforecasting/ManualUsuario_Version3.pdf).

**CUADRO 1.** Cambios de la herramienta de estimación de la demanda de antichagásicos y su relación con las limitaciones identificadas y cambios del contexto de la enfermedad de Chagas

	Versión 1.0 (2010)	Versión 2.0 (2012, anterior al taller presencial)	Versión 3.0 (2012, posterior al taller presencial)
Limitaciones identificadas o cambios de contexto	Lanzamiento de nuevas presentaciones de benznidazol Falta de datos epidemiológicos Ampliación de la recomendación técnica sobre el tratamiento de adultos con benznidazol		Ausencia de la opción de estimar benznidazol 12,5 mg para los casos de Chagas congénito a partir de la población de gestantes
Presentaciones	Benznidazol 100 mg Nifurtimox 120 mg	Benznidazol 100 mg **Benznidazol 50 mg** **Benznidazol 12,5 mg** Nifurtimox 120 mg	Benznidazol 100 mg Benznidazol 50 mg Benznidazol 12,5 mg Nifurtimox 120 mg
Tipos de fuentes de datos para cálculo	A partir de los datos epidemiológicos relativos a la enfermedad de Chagas (población general y prevalencia de la enfermedad)	A partir de los datos epidemiológicos **A partir del histórico de personas tratadas** **A partir del histórico de compras**	A partir de los datos epidemiológicos, **incluyendo la población de gestantes para el cálculo de la estimación de benznidazol 12,5 mg** A partir del histórico de personas tratadas A partir del histórico de compras
Variables de decisión	Programas especiales Ampliación del número de profesionales involucrados Ampliación de centros de salud que ofrecen el servicio de atención para la enfermedad de Chagas	Programas especiales Ampliación del número de profesionales involucrados Ampliación de centros de salud que ofrecen el servicio de atención para la enfermedad de Chagas **Ampliación del tratamiento de adultos (afecta apenas la cantidad de benznidazol 100 mg a ser demandada)** **Cálculos por mix de productos**	Programas especiales Ampliación del número de profesionales involucrados Ampliación de centros de salud que ofrecen el servicio de atención para la enfermedad de Chagas Ampliación del tratamiento de adultos (afecta apenas la cantidad de benznidazol 100 mg a ser demandada)
Otros	Cálculos por productos	(la posibilidad de estimación de más de un producto a la vez)	**Ajustes en la tabla de referencia de dosis del nifurtimox**

***Fuente:*** elaboración propia. Las partes en negrita son los cambios incorporados en las nuevas versiones de la herramienta.

El usuario de la herramienta puede iniciar su estimación a partir de la entrada de los siguientes datos: población del país y sus respectivos grupos etarios, histórico de personas tratadas en los últimos años por grupo etario, e histórico de compras de los medicamentos (compra o donación). El segundo paso es identificar cuáles serán los antichagásicos incluidos. Las tres presentaciones de benznidazol buscan atender diferentes grupos etarios, ya que el cálculo de la dosis se realiza con base en el peso medio del individuo. En este sentido, la herramienta ya apunta un cálculo de referencia del número medio de comprimidos por tratamiento según franjas etarias, a partir de un peso promedio (cuadro 2). Además, la herramienta permite la posibilidad de variar el peso promedio según las franjas etarias, lo que modifica la estimación de comprimidos por tratamiento.

**CUADRO 2.** Datos de referencia del número medio de comprimidos de benznidazol por tratamiento según franjas etarias

Grupo	Franja etaria (años)	Peso promedio (kg)	12,5 mg		50 mg		100 mg	
			Comprimidos por día	Comprimidos por tratamiento	Comprimidos por día	Comprimidos por tratamiento	Comprimidos por día	Comprimidos por tratamiento
Grupo I	0 a 1	8	4	240	-	-	-	-
	1 a 2	12,5	6	360	2	120	-	-
	2 a 5	25	-	-	4	240	2	120
	5 a 15	30	-	-	4	240	2	120
	15 a 20	50	-	-	-	-	3	180
	Adultos	80	-	-	-	-	4	240
Grupo II	0 a 1	8	4	240	-	-	-	-
	1 a 5	12,5	6	360	2	120	1	60
	5 a 15	30	-	-	4	240	2	120
	15 a 20	50	-	-	-	-	3	180
	Adultos	80	-	-	-	-	4	240

***Fuente:*** Iniciativa Medicamentos para Enfermedades Olvidadas, Organización Panamericana de la Salud, Médicos Sin Fronteras, 2013.

Es importante destacar, si se considera que para el momento del taller los países solo contaban con el histórico de uso de benznidazol en presentaciones de 100 mg, que la estimación por franjas etarias contiene un nivel mayor de complejidad de datos que la que el histórico de compras podrá responder. Los países que usaron como base este tipo de datos para la estimación de las necesidades de las presentaciones de 50 mg y 12,5 mg, lo hicieron sobre la base de otros criterios tales como proyección de la demanda futura y ampliación futura de cobertura de tratamiento para esas franjas etarias en sus servicios de salud.

Por otra parte, la herramienta considera dos opciones de franjas etarias para contemplar los diferentes contextos de los países y para aumentar la precisión de la estimación de los comprimidos de 12,5 mg, que tiene como población objetivo niños de 0 a 2 años de edad. En el Grupo I, los niños están divididos en cuatro franjas etarias (0 a 1, 1 a 2, 2 a 5 y 5 a 15 años), mientras en el Grupo II están divididos solo en tres franjas (0 a 1, 1 a 5 y 5 a 15 años). Como la presentación 12,5 mg incluye los casos de Chagas congénito, también se incorporó la posibilidad de estimación a partir de la población de mujeres embarazadas.

En un escenario considerado ideal, partiendo de la población del país y de sus grupos etarios, otro elemento para estimar la demanda es la identificación de la prevalencia por grupo etario para llegar a la estimación de una población seropositiva. A continuación, se debe estimar la capacidad de los servicios de salud para realizar el diagnóstico y ofrecer tratamientos (figura 1). Una síntesis del algoritmo para el cálculo de la demanda estimada (total de comprimidos) es el siguiente: número estimado de casos positivos × porcentaje de la capacidad de diagnosticar casos × porcentaje de la capacidad de tratar los casos diagnosticados × cantidad del producto seleccionado necesario para un tratamiento). Otros datos, considerados por la herramienta como variables de decisión, pueden afectar el porcentaje de la capacidad de diagnosticar nuevos casos.

Una vez consolidadas las estimaciones de demanda de benznidazol y nifurtimox presentadas después de la aplicación del ejercicio realizado durante el taller, se procedió a realizar un análisis más detallado de los datos obtenidos, específicamente en el caso del benznidazol. A partir de la población de América Latina estimada por la Comisión Económica para América Latina y el Caribe (CEPAL) en 2009 (n = 538 801 407, en Argentina, Belice, Bolivia, Brasil, Chile, Colombia, Costa Rica, Ecuador, El Salvador, Guatemala, Guyana, Guyana Francesa, Honduras, México, Nicaragua, Panamá, Paraguay, Perú, Surinam, Uruguay y Venezuela) y de su distribución por franja etaria, se calculó la proporción hipotética de personas infectadas con la enfermedad de Chagas por dicha franja, considerando la estimación disponible para la época de 7,6 millones (32) de personas infectadas en la región. Esta distribución posibilitó trazar un “escenario ideal” del número de personas con necesidad de tratamiento. Para los propósitos de estimación, se consideró la indicación de tratamiento para el 100% de los niños y adolescentes de 0 a 15 años diagnosticados como positivos.

Para el cálculo del “escenario ideal” también se consideró 65% del restante de la población, teniendo como base que aproximadamente el 30% a 40% de la población infectada evoluciona hacia la fase crónica de la enfermedad (32). Luego, a partir del total de comprimidos 12,5 mg (de 0 a 1 año de vida) y 100 mg (a partir de 1 año) estimado en el taller, se calculó la estimación de tratamiento por franja etaria respetando la distribución poblacional calculada a partir de la población total de la región, con base en el esquema padrón de tratamiento para cada grupo (cuadro 3). Por último, se calculó la proporción de tratamientos previstos en el taller en relación a la estimación de tratamientos para el “escenario ideal”.

## RESULTADOS

Catorce países endémicos de la región latinoamericana participaron de la etapa de presentación, validación y adaptación de la herramienta, con definición de una estimación consolidada de los tratamientos por franja etaria.

Aunque las estimaciones realizadas en el taller no tenían carácter oficial ni representaban una decisión final de compra por parte de los países, se logró consolidar el número aproximado de una posible orden de compra (cuadro 4)^6^. Los datos de entrada para el cálculo de la demanda más utilizados fueron la población del país y el histórico de compras. Solo un país utilizó el histórico de personas tratadas.

**CUADRO 3.** Estimativa de la proporción de tratamientos con benznidazol frente al escenario de las necesidades (“escenario ideal”) en América Latina

Franja etaria (años)	Distribución población total por franja etaria en AL	Proporción de cada franja etaria en relación a la población total de AL^a^	Distribución de la población infectada con Chagas en AL (“escenario ideal”) ^b^	Estimativa de la población elegible para el tratamiento (65% para las personas mayores de 15 años)^c^	Proporción de comprimidos por franja etaria (a partir del taller)	Tratamientos estimados (a partir del taller)^d^	Porcentaje de tratamientos con base en la estimación del taller en relación con el “escenario ideal”
< 1	7 561 391	0,0140	106 656	106 656	208 175 (comprimidos de 12,5 mg)	867	0,81
1 a 15	144 826 661	0,2688	2 042 835	2 042 835	1 058 707 (comprimidos de 100 mg)	8 822	0,43
15 a 20	49 940 905	0,0927	704 436	457 883	365 076 (comprimidos de 100 mg)	2 028	0,29
Adultos	336 472 450	0,6245	4 746 073	3 084 947	2 459 669 (comprimidos de 100 mg)	10 248	0,22

***Fuente:*** elaboración propia.

AL, América Latina.

a Población por franja etaria dividida entre la población total de AL según la CEPAL, 2009 (538 801 407).

b Población estimada con la enfermedad de Chagas en la región multiplicada por la proporción de franja etaria en relación a la población total en AL.

c Total de comprimidos de 100 mg estimados en el taller, distribuidos según la proporción de la población por cada franja etaria.

d Tratamientos estimados a partir del número de comprimidos por tratamiento establecido en el esquema de referencia (cuadro 1).

**CUADRO 4.** Valor consolidado de la estimación de demanda de los antichagásicos durante el taller en Brasilia, 2012

Medicamentos	Cantidad estimadas por los países y MSF para 2012-2013
Nifurtimox	774 947 comprimidos
Benznidazol 100 mg	3 041 670 comprimidos
Benznidazol 12,5 mg	208 175 comprimidos
Benznidazol 50 mg^a^	1 794 117 comprimidos

***Fuente:*** elaboración propia a partir de las estimaciones hechas por los países durante el taller realizado en Brasilia. Datos no oficiales.

MSF, Médicos Sin Fronteras.

a Durante la reunión, esta presentación no estaba disponible, aun así el cálculo fue realizado.

La estimación de la población elegible para tratamiento es de 5 692 321 personas (cuadro 4). Según el grupo etario, el total elegible para tratamiento estimado fue el siguiente: 106 656 (menores de un año), 2 042 835 (de 1 a 15 años), 457 883 (de 15 a 20 años) y 3 084 947 (adultos mayores de 20 años).

La demanda de comprimidos de benznidazol 50 mg fue estimada en 1 794 117 (cuadro 4) debido a que no estaba disponible la presentación de 50 mg al momento del taller. Se decidió expresar dicha cantidad en términos de comprimidos de 100 mg (897 059, resultado de dividir la cantidad estimada de comprimidos de 50 mg por dos) para adicionarla a la demanda estimada de esta presentación, comercialmente disponible. Así, la demanda total estimada para comprimidos de benznidazol 100 mg fue 3 938 728 (3 041 670 + 897 059).

Por otra parte, el histórico de ventas de comprimidos de benznidazol presentado por LAFEPE durante el taller para el período de 2008-2012 fue el siguiente: 528 800 (2008), 1 003 200 (2009), 404 600 (2010), 1 749 500 (2011) y 3 524 600 (2012). Se puede observar que los valores de demanda tuvo un aumento considerable a partir de 2011.

A partir de la estimación de comprimidos requeridos por los países durante el taller de 2012, fue posible establecer cuánto representa este cuantitativo en relación al número de tratamientos de adultos y niños (cuadro 3).

El número de tratamientos estimados a partir del número de comprimidos por tratamiento establecido en el esquema de referencia fue de 867 (en menores de un año), 2 042 835 (de 1 a 15 años), 2 028 (de 15 a 20 años) y 10 248 adultos mayores de 20 años) (cuadro 3). La proporción de tratamientos estimados frente al “escenario ideal” de necesidades para América Latina apunta que para los casos congénitos que demandarían la presentación 12,5 mg del medicamento benznidazol, la cantidad estimada representa 0,81% de los tratamientos necesarios para tratar esta franja etaria en la región. Para el grupo de niños y adolescente entre 1 a 15 años), el valor estimado es de 0,43%. Ya para los casos entre 15 a 20 años y adultos el valor estimado es de 0,29% y 0,22%, respectivamente.

Este cuantitativo representa la posibilidad de tratar menos de 1% de las personas con indicación para tratamiento con benznidazol.

## DISCUSIÓN

Entre los principales desafíos relacionados a la disponibilidad de medicamentos para enfermedades desatendidas está el número restringido de proveedores y la baja demanda de los productos. Esta dinámica requiere de esfuerzos e incentivos para asegurar que dicha oferta sea continua.

Por el lado de la demanda, el desafío para el acceso es asegurar que las tecnologías existentes puedan llegar a sus usuarios finales y pasar tanto por la distribución de medicamentos y su uso adecuado, como por la capacidad de los servicios en salud en detectar los casos de forma oportuna.

El objetivo del siguiente estudio consistió en desarrollar y describir una herramienta para estimación de la demanda de medicamentos antichagásicos y discutir su aplicación en un grupo de países de la región latinoamericana, a fin de demostrar la importancia de la consolidación de una demanda conjunta.

La metodología y los resultados presentan, sin embargo, algunas limitaciones. En relación a la metodología, el tipo de dato de entrada para el cálculo de estimación es una primera limitación ya que pocos países disponen de datos epidemiológicos para calcular la demanda a partir de encuestas poblacionales y de prevalencia de la enfermedad (32–34).

Una alternativa para superar esta brecha fue trabajar con datos epidemiológicos publicados, así como el histórico de compras o de personas tratadas. La limitación de los datos históricos al es que no refleja la cantidad efectiva del producto a ser utilizado ni la necesidad real existente. De la misma manera, al usar el histórico de compras o el número de personas tratadas, aquellos países que tienen previsto ampliar las actividades de diagnóstico y tratamiento encontrarán algunas limitaciones, una vez que las estimaciones futuras tienen como base las compras o tratamientos anteriores, por lo que es necesario tener en cuenta un porcentaje de aumento posiblemente arbitrario y sin reflejar la real demanda existente.

El cálculo de la proporción de tratamientos estimados en el taller en relación al llamado “escenario ideal” tiene como limitación el hecho de que, por opción metodológica, no se consideraron los contextos en que el nifurtimox es la opción de primera línea de tratamiento.

Por último, es importante también considerar que la intención de adquisición no refleja necesariamente la compra real ni tampoco la capacidad de implementación del tratamiento al paciente.

Las limitaciones apuntadas reflejan la importancia de trabajar en la construcción de datos confiables y tener un sistema de información robusto que permita contribuir en la planificación de la compra de medicamentos.

La herramienta no resolverá todos los desafíos que resultan de la falta de datos, pero es un esfuerzo colectivo y cooperativo diseñado entre los programas nacionales de control con apoyo de la OPS y la dinamización de organizaciones médicas y científicas como MSF y DND*i* de organización de la demanda, que ayudará en la planificación de la producción y contribuir para la disponibilidad del medicamento.

El esfuerzo de aplicar la herramienta ha permitido la identificación de brechas de información relacionadas con la enfermedad de Chagas y también la estimación de un cuantitativo aproximado de demanda basado en un fundamento teórico y parámetros de comparación entre países.

Durante el taller, la herramienta permitió a los países identificar el escenario de las necesidades y evaluar el escenario de las posibilidades, reveló de forma cuantitativa el camino a ser recorrido para ampliar el acceso a diagnóstico y tratamiento y se transformó en una potencial herramienta de incidencia política (*advocacy*) basado en evidencias.

La diferencia entre la previsión de tratamientos estimados en el taller, como intención de adquisición para 2012-2013, frente a las necesidades de tratamiento de las personas con la enfermedad de Chagas en América Latina es menor al 1%. Esta diferencia entre necesidad y demanda permite visualizar el tamaño del desafío para la expansión del acceso a tratamiento de la enfermedad de Chagas en la región y coloca a todos los actores involucrados a trabajar con urgencia en la búsqueda de respuestas y estrategias para lidiar con la complejidad de la enfermedad.

Los resultados apuntan a la urgente necesidad de una agenda integrada que busque soluciones concretas para la expansión de diagnóstico y tratamiento ofrecidos a los pacientes, así como a cambiar de manera radical el escenario del enfrentamiento a la enfermedad de Chagas en la región.

El desarrollo y el uso sistemático de herramientas de gestión de la demanda pueden jugar un papel clave en el apoyo al acceso a medicamentos para la enfermedad de Chagas. Existe una brecha significativa entre las previsiones de demanda de medicamentos y estimaciones actuales de las tasas de prevalencia.

Hace 107 años que se describió por primera vez la enfermedad de Chagas y aun es responsable por más de 10 000 muertes al año. A pesar de los compromisos y esfuerzos en pro de la ampliación de diagnóstico y tratamiento desde el año de su centenario de descubrimiento, es fundamental encontrar un nuevo abordaje para cambiar este cuadro mundial de negligencia.

### Declaración.

Las opiniones expresadas en este manuscrito son responsabilidad del autor y no reflejan necesariamente los criterios ni la política de la *RPSP/PAJPH* y/o de la OPS.
